# Biotransformation of Canola Feedstock Waste Using Brassica Pest Microbiome: Proof of Concept for Insects as Bioengineers

**DOI:** 10.3390/ijms26167715

**Published:** 2025-08-09

**Authors:** Avinash V. Karpe, Tom K. Walsh, Adam J. Carrol, Xue-Rong Zhou

**Affiliations:** 1 CSIRO Agriculture & Food, Clunies Ross Street, Canberra, ACT 2601, Australia; xue-rong.zhou@csiro.au; 2 CSIRO Environment, Clunies Ross Street, Canberra, ACT 2601, Australia; tom.walsh@csiro.au; 3 RSB/RSC Joint Mass Spectrometry Facility, Research School of Chemistry, Australian National University, Acton, ACT 2601, Australia; adam.carrol@anu.edu.au

**Keywords:** glucosinolates depletion, SCFA elevation, S-adenosyl methionine (SAM), methionine, cysteine and glutathione metabolism, sinapin and syringate degradation, tyrosine, tryptophan and phenylalanine metabolism

## Abstract

The toxicity of glucosinolate, isothiocyanate and sinapin limits canola meal’s use as non-ruminant animal feed. While monoculture microbial biorefining has been explored, the potential and capability of insect-associated microbiomes in this context remain underexplored. Herein, we extracted the gut and frass extracts from canola feeding larvae of Heliothis moth (HP), cabbage white (WCF) and cabbage looper (CL). Canola meal was fermented for one week with these extracts, followed by liquid chromatography–mass spectrometry (LC-MS)-assisted metabolomics analysis. Elevated branched-SCFAs 2-hydroxy butyrate and 3-hydroxy butyrate and propionate were observed in HP and WCF ferments, respectively. Aliphatic glucosinolates and sinapins showed ≥2-fold depletion in the HP and WCF frass ferments. In gut extract and frass-fermented canola meal, particularly of the HP group, tryptophan, tyrosine, and cysteine and glutathione metabolism were the most impactful pathways, aiding biogenic amine and branched-SCFA synthesis. S-adenosyl methionine (SAM) led salvaging, playing a key role in amino acid recycling via mercapturate metabolism, oxidative stress handling via the methionine and cysteine metabolism pathway, and sinapin metabolism through syringate degradation. These findings highlight the metabolic mechanism of brassica herbivore insect gut microbiome in detoxifying and adding value to canola meal. Such microbial communities have the potential to upcycle canola meal into a nutrient-rich feed additive with gut-health-promoting properties.

## 1. Introduction

Canola is a major Australian broadacre crop, producing 7.5 million tonnes in 2023–2024 [1]. Canola meal constitutes up to half of the total canola seed biomass (*w/w*) and is the major waste product of oil production [2]. Although it is the second most used animal feedstock after soybean meal [2], its use as a feedstock is limited due to glucosinolates, thiocyanates, isothiocyanates, and sinapins [2]. Generally, the pre-conditioning of seeds at 80–90 °C [3], or, in some cases, 135 °C [2], minimises the myrosinase activity. The enzyme catalyses the otherwise non-reactive glucosinolates to more toxic intermediates such as thiocyanates, isothiocyanates and nitriles, among others [2]. This combination of sinapins (1%), low-level thio/isothio-cyanates, and the low energy density of non-dehulled canola meal compared to soybean meal has been shown to compromise growth by up to 5.7% in non-ruminant animals such as poultry [4] and digestibility in swine [5].

Microbial bioprocessing is a good method to address these issues and achieve a biotransformation rather than degradation [6]. Recent studies conducted with black soldier fly (BSF) and other potential candidates have indicated the role of insects and their gut microbiome for biomass conversion [7,8,9]. The BSF larvae are able to increase lignocellulose degradation to 26% with respect to 4% by composting over 14 days, with gut bacteria such as *Corynebacterium*, *Brevibacterium*, *Dysgonomonas*, *Prevotella*, and *Cellulomonas* playing key roles [8]. Along similar lines, lepidopteran generalist herbivore pests such as Heliothis moth larvae are known to digest biomass while resisting the toxicity of glucosinolates, cyanates, and sinapins when feeding on Brassica crops such as canola [10,11,12]. Broadly, glucosinolate induction creates downregulated larval hydrolase, oxidoreductase and transferase activities, thus creating oxidative stress within *Helicoverpa* and *Spodoptera* guts [13,14]. The larval detoxification enzymes such as carboxylesterases within the *Helicoverpa* midgut have been shown to counter this toxicity [10]. However, the presence of key microbial communities such as *Pantoea*, *Lactococcus* and *Enterobacteriacae* increased the polyphenol oxidase activity, and therefore, larval–plant interaction and responses [10]. Gut microbiota suppression studies of cabbage stem fly beetle have indicated the role of bacteria such as *Pantoea*, *Acinetobacter* and *Pseudomonas* in isothiocyanate metabolism and detoxification [12]. Particularly, *Pantoea* were observed to be the key isothiocyanate degrading species in the study.

While this property has been explored in multiple studies that consider these herbivores as pests on the brassica plants [12,13,14,15,16], its application in commercial biomass transformation is limited. Our previous study on BSF larvae highlighted their metabolic behaviour and gut microbial role during plastic degradation. In particular, the induction of biotic stress caused by the toxic effects elevated the metabolic pathways of Vitamin B6 and butanoate metabolism [7]. Furthermore, the application of high-throughput platforms such as metabolomics has the potential to yield a broader understanding of biochemical mechanisms, as shown in previous studies on gut interactomics [17,18] and larval herbivory [9,16]. The functional aspect of metabolomics can deliver excellent complementation to the predictive nature of genomic analyses, which have been shown in insect gut processes [8,12,13,19]. In the current study, we used this background to understand the mechanism of canola meal transformation under in vitro conditions using larval gut extracts and frass (containing microbial communities). We conducted a comparative analysis of cabbage looper, Heliothis moth, and cabbage white larval gut and frass extracts with canola meal as the target biomass. While most studies we cited relied on transcriptomic prediction in their studies, we utilised liquid chromatography–mass spectrometry (LC-MS)-assisted metabolomics to assess the functional aspects of these bioconversions. These included glucosinolate depletion, coupled with an increase in short-chain fatty acids (SCFAs). The metabolomics assessment also provided insights into the mechanism of isothiocyanate, thiocyanate and sinapin metabolism, as well as the key pathways involved in this bioconversion.

## 2. Results

The current study was performed to assess two hypotheses: (1) whether the gut and frass extracts from actively feeding herbivore larvae on canola plant can degrade/transform toxic components such as isothiocyanates and sinapins, and (2) if so, then what is the biochemical mechanism of this transformation? While methods of genomic analysis such as whole-genome sequencing and 16S rRNA genomic sequencing would have allowed us to further explore these questions, to avoid descriptiveness in the study, only metabolomic assessment was kept in the study as a proof-of-concept.

In this context, important outputs such as short-chain fatty acids were considered as indicators of microbial activity. The microbial effects were assessed through glucosinolate, isothiocyanate and sinapin depletion. Finally, the mechanism of countering toxicity stress and biomass transformation was assessed by comparative metabolic behaviour and pathway impacts.

### 2.1. Quality Control Analysis

The metabolomics analysis indicated a presence of ca. 4301 metabolic features across both negative and positive polarities of mass spectral scans. Post-filtration data indicated the presence of 1112 metabolites, of which one-way ANOVA analysis identified 658 as statistically significant (false discovery rate (FDR)-adjusted *p*-value ≤ 0.05) metabolites. The relative standard deviation for internal standards ^13^C_4_-Succinic acid and ^13^C-Phenylalanine was observed at 12.73 and 11.19%, respectively. The percent-RSD for QC metabolites is presented in Table 1 below.

### 2.2. Microbial Activity: SCFA Production and Glucosinolate Depletion

In addition to the broader metabolic activity, the efficiency of canola meal biotransformation by the insect microbiome was assessed via short-chain fatty acid (SCFA) production, as well as the depletion of antinutrients such as glucosinolates and sinapic acid derivatives.

**Figure 1:**
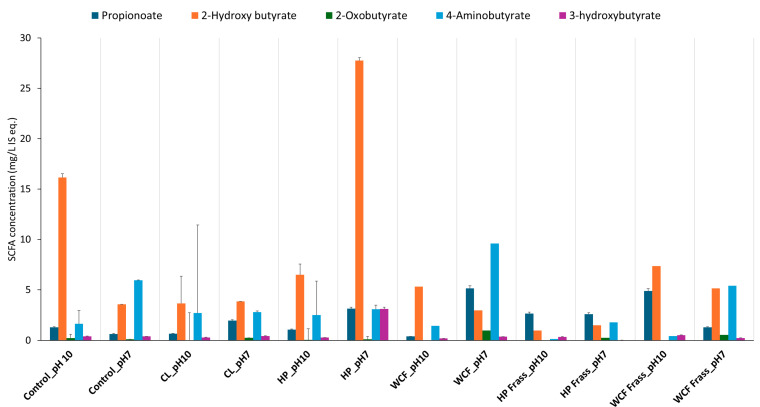
SCFA distribution after microbiome fermentation from homogenised whole insects and frass of canola feedstock at pH 10 and pH 7 with tested larvae. Note: the terms CL, HP and WCF indicate larvae of cabbage looper, Heliothis moth and cabbage white butterfly, respectively. Note: values on the Y-axis indicate the relative concentration of detected metabolites with respect to the internal standard ^13^C-phenylalanine. Therefore, they are expressed as mg/L internal standard equivalent (IS eq.).

The data analysis indicated the presence of 6 SCFAs/ branched SCFAs across all the samples. Overall observations indicated that the fermented samples at pH 7 generated higher levels of SCFAs compared to pH 10 (Figure 1).

**Figure 2:**
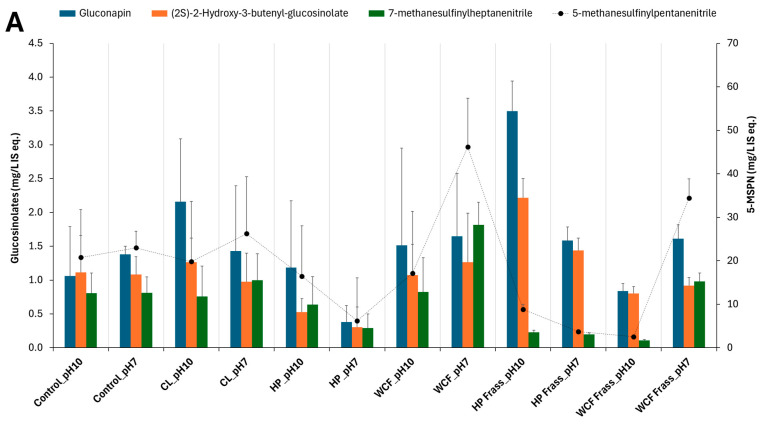

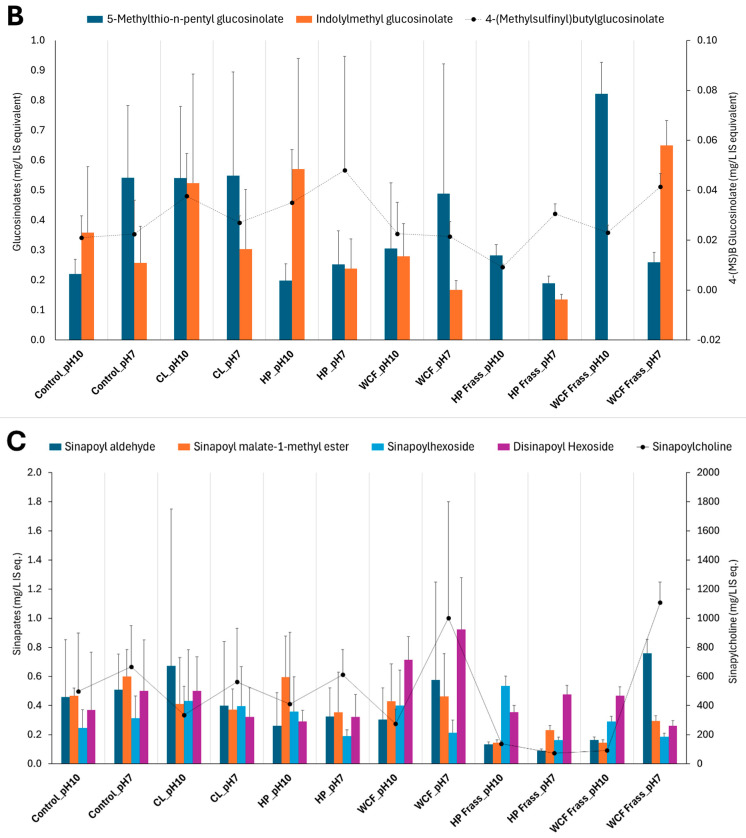
Glucosinolate and sinapin distribution in larval microbiome fermentation of canola feedstock at pH 10 and pH 7. Note 1: the terms CL, HP and WCF indicate cabbage looper, Heliothis moth and white cabbage moth, respectively. (**A**,**B**) show the glucosinolates and their degradation products such as nitriles; (**C**) represents the sinapins and sinapic acid derivatives. Note 2: to accommodate the different compounds of the same group of metabolites, the secondary axis is used. Here, the secondary axis refers to the levels of 5-methylsulfinyl pentanitrile (5-MSPN) (**A**), 4-(methylsulfinyl) butyl glucosinolate (**B**) and sinapoyl choline (**C**). Note 3: Values on the Y-axes indicate the relative concentration of detected metabolites with respect to the internal standard ^13^C-phenylalanine. Therefore, they are expressed as mg/L internal standard equivalent (IS eq.).

Similar to the SCFA production, pH 7 was observed to be create a greater depletion of glucosinolates, sinapins, and their derivatives. Specifically, the frass fermentation resulted in higher degradation compared to the gut content fermentation (Figure 2).

It was also noted that the HP microbiome fermentation of canola meal by both gut and frass not only showed relatively more elevated SCFAs and branched SCFAs but also resulted in a greater depletion of glucosinolates, sinapins, and their derivatives. Particularly, glucosinolates such as gluconapin (3.6 fold, Figure 2A) and nitriles (2.7–3.7 fold) showed a considerable depletion in HP gut ferment at pH 7. Similarly, HP frass ferment at pH 7 showed an almost twofold depletion of glucobracissin (indolylmethyl glucosinolate, Figure 2B), and sinapins such as sinapylcholine (nine fold, Figure 2C) and sinapoyl aldehyde (>fivefold), with respect to the control. The only exception was sinapoyl choline, which showed a greater depletion during the WCF frass fermentation (Figure 2C).

### 2.3. pH Differences Showed No Statistically Significant Metabolic Differences

The samples were fermented at pH 7 and pH 10 to assess whether there was a differential expression of metabolic pathways at those values. pH 10 was selected to mimic the natural conditions of sections of the larval gut, as it has been shown previously that the gut region of lepidopteran larvae can become very alkaline (pH 8–12), although the frass tends to be slightly acidic (pH 4–7) [20]. It has also been suggested that the high alkaline pH promotes the degradation of complex compounds such as tannins in the larval midgut [10]. Interestingly, a study conducted on waste-activated sludge showed that the pre-treatment of the sludge by maintaining it at pH 10 for some time before fermentation increases the biodegradation and methane production [21].

**Figure 3:**
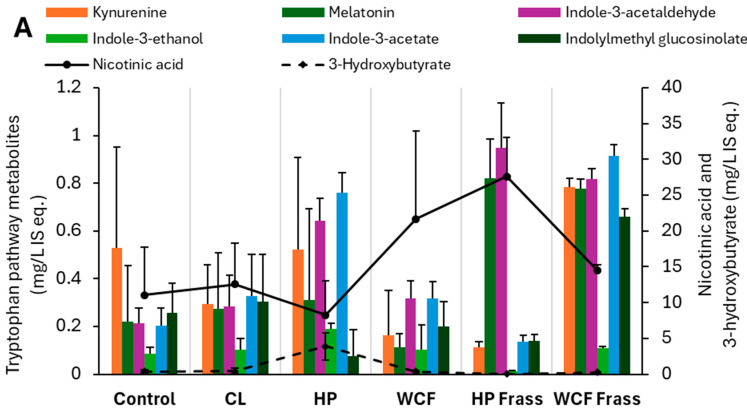

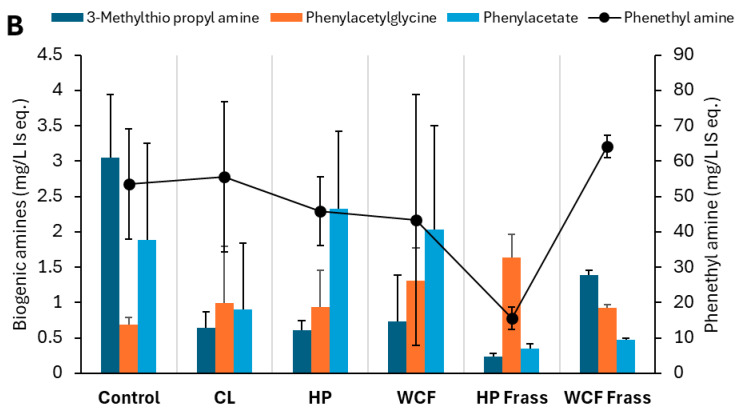
Key metabolites observed from the pH 7 ferment dataset indicating (**A**) glucobracissin (indolylmethyl glucosinolate) degradation via tryptophan metabolism, and (**B**) biogenic amine biosynthesis and metabolism via phenylalanine metabolism. Note 1: values on the Y-axis indicate the relative concentration of detected metabolites with respect to the internal standard ^13^C-phenylalanine. Therefore, they are expressed as mg/L internal standard equivalent (IS eq.). Note 2: to accommodate the different compounds of the same group of metabolites, the secondary axis is used.

The assessment of control samples in the current study indicated that almost none of the central carbon metabolites were altered in a statistically significant manner between neutral and alkaline pH values. Of the 49 significant metabolites (FDR-adjusted *p*-value ≤ 0.05) that were observed to be statistically different between pH 7 and pH 10, most consisted of secondary metabolites such as polyphenols (Supplementary Figure S1, Supplementary Table S1), which were released due to a possible minor degradation caused by NH_4_OH at pasteurisation temperature of 72 °C. The impact of alkaline treatment at elevated temperatures towards polyphenol release from grape pomace on biomass has been shown previously [22]. However, in the current study, the initial alkalinity was not observed to create significantly different metabolic profile, compared to pH 7 (Figure 3). It was also observed that, at the end of the fermentation, all the test samples had pH range of 4.9–7.3, indicating considerable acidification. This could be the key reason that the differences were not statistically significant between pH 7 and pH 10 ferments.

### 2.4. Metabolic Transformation During the Canola Meal Fermentation

The behaviour of 658 statistically significant metabolites was assessed with the PCA and PLS-DA for better discrimination (components = 8, cross validation = 5-fold CV, 95% confidence interval) (Supplementary Figure S2). The prediction accuracy measured with 100 permutations for separation distance indicated the model to be statistically significant (PLS-DA empirical *p*-value < 0.01; PermANOVA F-value = 0.5919, *p*-value = 0.001).

The data were subjected to a pathway analysis toolbox of Metaboanalyst 5.0, against the *Bombyx mori* species metabolomic reference. The analysis indicated that 45 metabolic pathways were statistically significant (FDR adjusted *p*-value ≤ 0.05) (Supplementary Table S2), with the top 15 (Impact ≥ 0.4) shown in Table 1. As glucosinolate degradation and SCFA production were primarily observed in the pH 7 ferments, the behavioural pattern of the statistically significant metabolites from the key pathways (Table 2) was assessed against the pH 7 ferments. Primarily, two types of metabolisms, i.e., sulphur and amine metabolisms, were seen.

#### Species Comparison

The metabolic output of species was compared using univariate analyses. The groups compared included (1) CL vs HP, (2) WCF vs HP, (3) CL vs WCF, (4) HP vs HP Frass, (5) WCF vs WCF Frass, and (6) HP Frass vs. WCF Frass. Within the gut extract fermentation, significant differences in metabolic profile (FDR adjusted *p*-value ≤ 0.05) were observed only for Group 2: WCF vs. HP (Table 3). Particularly, indole-3-acetate, indole-3-acetaldehyde and tetradecanoic acids were elevated in HP gut extract ferment. No statistically significant difference was observed for CL vs WCF group.

Comparison between ferments of gut extract and frass showed 36 and 22 metabolites being elevated in gut extract ferments of HP and WCF, respectively, when compared to their respective frass ferments (Dataset S1, Supplementary Materials).

### 2.5. Aromatic Glucosinolate Degradation Results in Nicotinate, SCFA Biosynthesis, and Mercapturic Acid Pathway Led Biogenic Amines Synthesis

In the tryptophan metabolism, glucobracissin (indolylmethyl glucosinolate) degradation was observed to happen through indole intermediates. In HP gut ferment, indole-3-acetaldehyde, indole-3-ethanol and indole-3-acetate were elevated. On the other hand, melatonin and indole-3-acetaldehyde showed elevation in HP frass ferment. However, in the gut ferment, an increased 3-hydroxy butyrate indicated a likely metabolism of indoles to pyruvate metabolism via an acetyl-CoA intermediate. While, in the frass ferment, these intermediates appeared to be diverted to nicotinic acid metabolism, as evidenced from a considerable increase of nicotinic acid (Figure 3A).

### 2.6. Gut Microbe-Driven Metabolism Plays a Key Role in Oxidative Stress Handling During Glucosinolate and Sinapin Degradation

**Figure 4:**
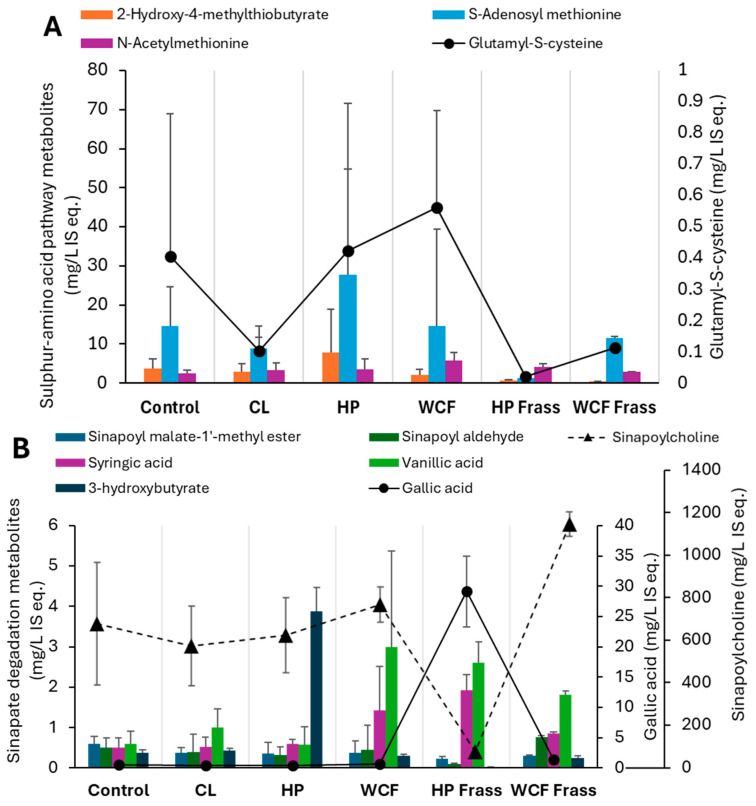
Key elevated and depleted metabolites of (**A**) cysteine and methionine metabolism and (**B**) Sinapic acid degradation, observed in the pH 7 ferments of canola meal. Note 1: Values on Y-axis indicate the relative concentration of detected metabolites with respect to the internal standard ^13^C-phenylalanine. Therefore, they are expressed as mg/L internal standard equivalent (IS eq.). Note 2: For accommodating the different compounds of the same group of metabolites, secondary axis is used.

Besides myrosinase, the enzymes such as isothicyanate hydrolases directly produce amines, while glutathione S-transferase activities first produce ITC-γ-glutamylcysteine and other intermediates (Figure 4A), and biogenic amines (Figure 4B) [25]. Elevated S-adenosyl methionine (SAM) and γ-glutamyl-S-cysteine indicated amine salvaging through methionine metabolism, leading to glutathione synthesis via intermediate (Figure 4A). This was particularly observed in the HP gut where both SAM and 2-hydroxy-4-methylthiobutyrate (HMTB) elevations indicated methionine recovery, tight regulation of sulphur and methyl balance under oxidative stress by the gut microbes.

## 3. Discussion

We used high-throughput metabolomics to assess the biochemical activities of gut and frass microbiomes during canola meal fermentation. *Helicoverpa zea* and Cabbage white (WCF) larvae feeding on BT-cotton and cabbage, respectively have shown the enriched gut microbiota for modulation of their environment to counter biological and environmental stresses [11,19,26]. The recent 16S rRNA-based amplicon study of the rapeseed pest, cabbage stem flea beetle [12] indicated the importance of microbiome members such as *Pantoea* spp. towards enabling the isothiocyanate degradation. The current study showed that the gut microbiome of brassica herbivore insects play an important role in the biomass degradation of canola meal. Particularly, it played a key role in acidification of all tested ferments, as reported previously [26] during the glucosinolate and sinapin transformation. Myrosinase induced metabolism of glucosinolates in the plant cell is well known, resulting into production of indoles, isothiocyanates (ITC), epithinitriles and nitriles [27,28,29]. However, the fate of these products especially in herbivore larval gut, is relatively less known. Biogenic amines such as methylthio propyl amine play an important role in the glucosinolate catabolism [30]. However, the fate of these amines is not widely discussed. The mercapturic acid pathway has been discussed within human and animal models in relation to xenobiotic metabolism [30,31], but to a limited degree from a glucosinolate degradation perspective. In the current study, microbial metabolism likely drove the mercapturic acid pathway to deaminate the biogenic amines through methionine, and cysteine and glutathione metabolism pathways (Refer Section 2.6, Figure 4A).

The production of SCFAs and branched-SCFAs (Containing 6 or less carbons) are considered a good marker for the gut microbiome activity [18,32,33]. They are produced by the gut bacteria during the anaerobic fermentation of non-digestible carbohydrates and resistant starch. Furthermore, they modulate the gut microbiome against numerous stresses that gut environment is exposed to [32]. The inhibition study on *Spodoptera litura* by azadirachtin enriched diet showed a depletion of fatty acids and key bacterial species such as *Faecalibacterium* and *Lactobacillus* upon 2 mg/kg azadirachdin supplementation [34]. In *Bombyx mori*, the artificial diet feeding has shown to increase the SCFA producing bacteria such as *Weissella* and *Lactobacillus* [35]. Diet increasing the SCFAs have shown to produce some antifungal effects amongst insect larve [36,37,38]. Particularly, 3-fold increase of propionic acid secretion in *Beauveria* infected triatomine bug indicated the importance of SCFAs in immune response [39]. In *Drosophila*, bacterial generated propionic acid has shown to induce olfactory responses through Or30a^+^ and Or94b^+^ olfactory sensory neurons, improving larval survival [40]. A further microbiome genomic analysis is expected to help understand the role of specific HP and WCF larval microbiome that contributes to SCFA production, and other functional aspects.

**Figure 5:**
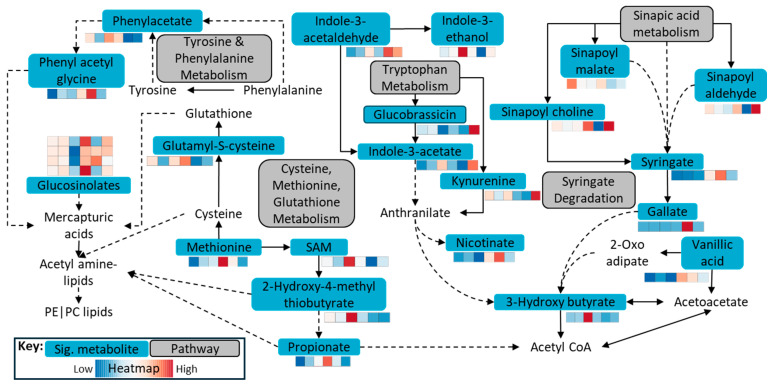
Key metabolic pathways observed during the fermentation of canola meal by the herbivore gut microbiome. Note: The heatmap represents, from left to right, Control—CL—HP—WCF—HP Frass—WCF Frass, displaying elevated or depleted statistically significant (FDR adjusted *p*-value ≤ 0.05) metabolites from the key pathways.

Xenobiotic degradation and metabolism was one of the key microbially driven pathways in WCF larvae [19]. On the other hand, metabolism of carbohydrates, lipids and 5-hydroxytryptamine were key metabolic pathways in *Helicoverapa* larval gut [41] with non-glucosinolate feed. However, the current study indicated that when the microbiome was applied to fermentation, metabolism of aromatic amino acids and sulfur-containing amino acids played a much significant role during glucosinolate, isothiocyanante and sinapin degradation (Figure 5). This was in line with the observation that sinigrin induced oxidative stress significantly upregulated the glutathione metabolism and oxidative phosphorylation in HP larvae [13].

### Limitations of This Study

The current study provided a considerable insight into the application of insect gut microbiome in the biomass processing. However, as it was a proof-of-concept, we acknowledge the limitations of the study. Firstly, the stage of larvae was not considered in this study, which may play a considerable role in gut microbial spread within the herbivore gut [41], and the resultant metabolism. Furthermore, the genomics, metaproteomics and lipidomic works will be needed to confirm the metabolomics outputs we presented in this study.

## 4. Materials and Methods

### 4.1. Canola Feedstock Treatment

Canola feedstock was procured internally within CSIRO. Larvae feeding on podding canola (canola growth stage 7) were collected from CSIRO Boorowa Agricultural Research Station (Week 1, November 2023). The larvae were collected as wild-type specimens in their environmental feeding condition directly from the canola plants grown in an open field paddock. The larvae were collected before the seasonal application of a pesticide treatment on the paddock. This ensured that the pesticides were not the impacting factors in measuring larval biochemistry. The collected larvae were stored in 50 mL Falcon tubes on ice to minimise metabolic activity and transport stress. Before processing, the larvae (*n* = 5) were rinsed with 0.9% saline, followed by 2 rinses with sterile distilled water in a sterile 2 mL centrifuge tube (round bottom, Eppendorf Pty Ltd., Sydney, NSW, Australia). For frass collection, larvae were kept in sterile tubes containing fresh canola leaves collected from the paddock. Larve were allowed to feed for 2 days under room-temperature conditions. Frass was collected after a 2-day incubation, vortex homogenised, and stored at −80 °C until analysed. The larvae were then transferred to sterile 2 mL centrifuge tubes (Eppendorf South Pacific, Macquarie Park, NSW, Australia). Sterile Luria Bertini (LB) broth (Ca. No. 12780052, Thermo Fisher Scientific, Melbourne, VIC, Australia), 1 mL, was added to these tubes. Two sterile stainless-steel beads (3 mm diameter, Cat. No. BMSD113328, Merck Life Sciences, Bayswater, VIC, Australia) were added to each tube, and larvae were homogenised through vortexing at 1000 rpm (Fastprep-96, MP Biomedicals, Irvine, CA, USA) to obtain gut extract.

**Figure 6:**
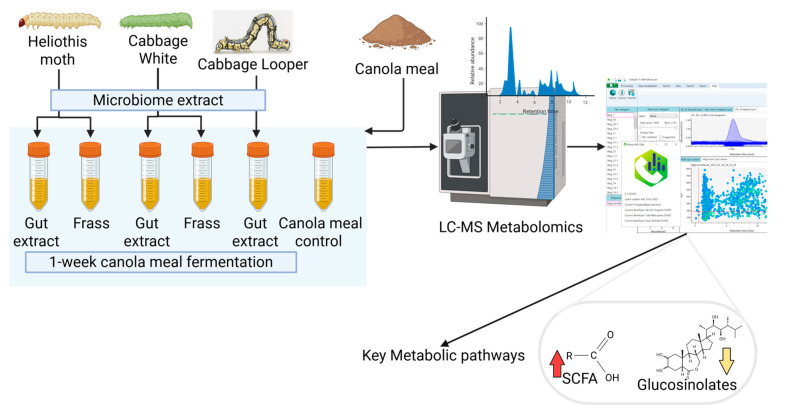
General schematic of canola meal fermentation by the herbivore gut and frass microbiome extract. Created in BioRender. Karpe, A. (2025) https://BioRender.com/ose7tp6 (accessed on 4 August 2025).

Canola meal, 1 g, was added to 20 mL sterile LB broth in a 50 mL tube (Code: 31301-BD, McFarlane Medical, Ringwood, VIC, Australia) and resterilised through pasteurisation (72 °C/30 min). The growth medium was cooled to room temperature, and 0.2 mL larval gut extract and frass homogenates were added to individual culture tubes (Note: due to the size of the cabbage looper larvae, frass could not be collected). To compare the fermentation and to mimic the conditions of the larval gut [20], one set each was fermented in a pH 7 and pH 10 medium (pH 10 adjusted by addition of NH_4_OH (Cat. No. 338818, Merck Life Sciences, Bayswater, VIC, Australia)), respectively. Both the sets were incubated 25 °C for 1 week, followed by immediate cooling to 4 °C. Slurry (1 mL) was pipetted from the culture and immediately stored at −80 °C to cease metabolism. Metabolomics analysis of the frozen samples was performed to identify the mechanism of canola feedstock bioconversion and quantify the biological transformation of glucosinolates and their degradation products (Figure 6). SCFA increase and the depletion of glucosinolates, isothiocyanates and sinapins were measured as the markers of canola meal detoxification. Furthermore, metabolome analysis was performed to understand the biochemical mechanism of fermentation, as indicated in Figure 6.

### 4.2. Metabolomics Sample Preparation and Analysis

Briefly, 100 µL of slurry was placed into a fresh 2 mL centrifuge tube and prepared for analysis as previously reported [18]. First, 450 µL of ice-cold ethanol: methanol (1:1), containing 1 µg/mL of internal standards (^13^C_4_-succinic acid, ^13^C-phenylalanine, both Cambridge Isotope Laboratories, Tewkesbury, MA, USA) was added to 100 µL slurry, followed by 100 µL ice cold MilliQ water. The mixture was then vortexed for 10 minutes at 1400 rpm/4 °C (Thermomixer, Eppendorf) and centrifuged at 14,000 g/2 min. The supernatant was transferred to an EMR lipid extraction cartridge on a vacuum manifold (Agilent Technologies, Mulgrave, VIC, Australia). The aqueous phase was collected in a fresh tube by applying a constant vacuum (2 inHg). The cartridge was rinsed with 200 µL ethanol: methanol: water (1:1:2) to elute any aqueous metabolites. The aqueous eluate was then dried under nitrogen and resuspended in 200 µL ice-cold acetonitrile: water (8:2). The mixture was vortexed at 1400 rpm/4 °C for 1 h on a thermomixer, followed by centrifugation at 14,000 g/2 min, and the supernatants were transferred to LC-MS vials for analysis.

For LC-MS analysis, 2 µL of the abovementioned supernatant was injected through an ACQUITY UPLC^®^ BEH Amide column, 150 × 2.1 mm, 1.7 µm ID (Part 186004802, Thermo Fisher Scientific, Melbourne, VIC, Australia) on Vanquish Liquid Chromatograph (LC), coupled with a QExactive Plus mass spectrometer (MS) (Thermo Fisher Scientific, Melbourne, VIC, Australia). The LC flowrate was maintained at 0.25 mL/min. Solvents A and B were acetonitrile: 10 mM ammonium formate (10:90), and acetonitrile: 10 mM ammonium formate (90:10), both pH 9, and they contained 5 µL medronic acid. The gradient for Solvent B was 95% (0–3 min.), 78% (8 min.), 30% (15 min.), and 95% (20 min.). The column was conditioned with 95% Solvent B for further 2 min. The MS analysis was performed in both negative and positive modes. The parameters (negative/positive) were spray voltage (2.5 kV/3.5 kV), capillary temperature (262 °C/262 °C), sheath gas (50/50), auxiliary gas (12.5/12.5), spray current (100/100), probe heater (425 °C/425 °C) and S-Lens RF level (50/50). A full scan was performed within the 60–750 m/z range, with the resolutions of 70,000 and 17,500 at MS1 and MS2 levels, respectively.

### 4.3. Statistical Analysis

The data obtained from LC-MS were processed on the MS-DIAL 5.0 platform [42,43], with the indicated settings (https://github.com/respiratory-immunology-lab/metabolome-lipidome-MSDIAL (accessed on 10 June 2025)).

The quality control (QC) metabolite mix was processed on the LC-MS system between every 10 samples. The QC mix contained 1 µg/mL each of pyruvic acid, serine, maleic acid, succinic acid, asparagine, salicylic acid, citrulline, glucose, citric acid, and tryptophan (all Sigma Aldrich, Alexandria, NSW, Australia).

Metabolite features with signal-to-noise (S/N) ratio ≥ 10, and match factor ≥ 70% with respect to the mass spectral database were used as data filtration thresholds. The filtered data were normalised against ^13^C-phenylalanine on Microsoft^®^ Excel (Microsoft^®^ Excel^®^ for Microsoft 365 MSO (Version 2507 Build 16.0.19029.20136) 64-bit).

This normalised data were then imported to MetaboAnalyst 6.0 for processing. Missing values for metabolite features were replaced by 1/5 of the positive value, followed by sample normalisation through the pooled sample group, and the auto scaling of the data. The normalised dataset was further subjected to univariate (one-way ANOVA, biomarker analysis), advanced significance (Significance of Microarray), and multivariate (principal component (PCA) partial least square-discriminant (PLS-DA)) analyses. Furthermore, the data were also applied to metabolic enrichment and pathway analysis toolboxes.

## 5. Conclusions

In this proof-of-concept study, the ability of the gut microbiome from brassica pest herbivores to bio-transform canola meal was assessed. We used LC-MS-based metabolomics to assess the biochemical changes that occurred during the fermentation. Among all the studied samples, the highest glucosinolate and sinapin depletion and SCFA increase occurred in HP gut and frass at pH 7 ferments. Of the 45 statistically significant metabolic pathways, aromatic amino acid and sulphur-containing amino acid metabolism were most prominent, countering toxicity-related stress. The metabolomic assessment indicated that the gut microbes of brassica pest herbivores drove considerable glucosinolate recycling and SCFA and biogenic amine production, thus decreasing the isothiocyanate toxicity for the host. Overall, we confirmed that the gut and frass microbes from canola-feeding herbivore insects can be harvested for the bio-transformation of agricultural wastes such as canola meal. After fermentation, the microbial extracts are not only able to detoxify the feedstock but also add compounds with nutraceutical benefits as food/feed, particularly within monogastrics such as poultry, pork, fisheries, and humans. The study also elaborated the metabolic pathways that are utilised to achieve this bio-transformation. The output of this study, post-scale up, is that microbial fermentation has the potential to increase the value-addition of agro-wastes such as canola meal for veterinary feedstock.

## Figures and Tables

**Table 1:** Quality control metabolites variation.

Metabolite Name	RSD (%)
Pyruvic acid	15.19
Serine	31.80
Maleic acid	16.79
Succinic acid	10.75
Asparagine	13.74
Salicylic acid	34.92
L-Citrulline	17.17
D-Glucose	15.69
Citric acid	15.11
Tryptophan	17.19

**Table 2:** Key pathways among the observed impactful pathways (FDR-adj. *p*-value ≤ 0.05) during the canola meal fermentation, as analysed using the pathway analysis toolbox of Metaboanalyst 6.0.

Metabolic Pathway	Hits/Total Compounds	FDR	Impact
Tyrosine metabolism	10/29	2.77 × 10^−10^	0.67
Pentose phosphate pathway	10/24	1.09 × 10^−08^	0.40
Alanine, aspartate and glutamate metabolism	14/21	3.33 × 10^−08^	0.98
Glycine, serine and threonine metabolism	9/30	9.77 × 10^−08^	0.73
Glutathione metabolism	8/26	2.21 × 10^−07^	0.49
Ascorbate and aldarate metabolism	2/9	1.47 × 10^−06^	0.52
Citrate cycle (TCA cycle)	8/20	2.72 × 10^−06^	0.47
Tryptophan metabolism	8/29	4.34 × 10^−06^	0.53
Arginine biosynthesis	9/13	0.0003	0.80
Phenylalanine metabolism	6/8	0.0004	0.74
Histidine metabolism	4/9	0.0028	0.4
One carbon pool by folate	9/23	0.0028	0.44
Vitamin B6 metabolism	3/8	0.0045	0.50
Cysteine and methionine metabolism	11/34	0.0048	0.48
Riboflavin metabolism	1/4	0.0129	0.5

Note 1: The terms hit/total compounds indicate the number of metabolites detected during the analysis, out of the total metabolites in the pathway. See Supplementary Table S2 for more information. Note 2: The pathway impact measures the significance of an individual pathway with respect to the whole metabolome. It is calculated as the sum of the importance measures of the matched metabolites normalised by the sum of the importance measures of all metabolites in each pathway. For further reading on this aspect, please refer to previous works [23,24].

**Table 3:** Metabolites with significantly (FDR adjusted *p*-value ≤ 0.05) elevated metabolites in the HP gut extract canola meal ferment compared to WCF ferment.

Metabolite	log_2_FC HP_(vs. WCF)_|FDR	log_2_FC HP_(vs. CL)_|FDR	Log_2_FC HP_(vs. HP Frass)_|FDR	Log_2_FC WCF_(vs. WCF Frass)_|FDR
FA 18:3 + 2O	−17.62|0.0080	−17.29|0.1343	NA	NA
D-Mannose-6-P	1.79|0.0080	0.61|0.0717	1.72|0.0237	NA
Indole-3-acetate	1.26|0.0080	1.21|0.0028	2.49|0.0084	−1.53|6.04 × 10^−5^
Tetradecanoic acid	2.29|0.0243	0.22|0.634	NA	−1.12|0.0004
6-Methoxyluteolin	1.37|0.0276	0.0276	NA	NA
Indole-3-acetaldehyde	1.02|0.0497	1.18|0003	NA	−1.37|0.0002

Note: A detailed list is provided in Dataset 1 of Supplementary Materials. The term FDR refers to FDR adjusted *p*-value.

## Data Availability

The original contributions presented in this study are included in the article/Supplementary Material. Further inquiries can be directed to the corresponding author.

## Supplementary Materials

The following supporting information can be downloaded at: https://www.mdpi.com/article/10.3390/ijms26167715/s1.

## Abbreviations

The following abbreviations are used in this manuscript:

HP	Heliotis moth
WCF	Cabbage white
CL	Cabbage lopper
SCFA	Short-chain fatty acid
SAM	S-adenosyl methionine
LC-MS	Liquid chromatography–mass spectrometry
CSIRO	Commonwealth Scientific and Industrial Research Organisation
QC	Quality control
S/N	Signal-to-noise
ANOVA	Analysis of variance
PCA	Principal component analysis
PLS-DA	Partial least square-discriminant analysis
FDR	False discovery rate
RSD	Relative standard deviation
5-MSPN	5-methylsulfinyl pentanitrile
ITC	Isothicyanate
HMTB	2-hydroxy-4-methylthiobutyrate
FAA	fatty acid amides
GLP-1	Glucagon-like peptide 1
