# Supplementary material for: Biotransformation of Canola Feedstock Waste Using Brassica Pest Microbiome: Proof of Concept for Insects as Bioengineers
Source: Int J Mol Sci. 2025 Aug 9;26(16):7715. doi: 10.3390/ijms26167715 (PMC12386942; doi:10.3390/ijms26167715)
Supplement: Supplementary file 1 [file ijms-26-07715-s001.zip › ijms-3758977_AK_V2_Supplementary Materials.pdf]

Supplementary materials

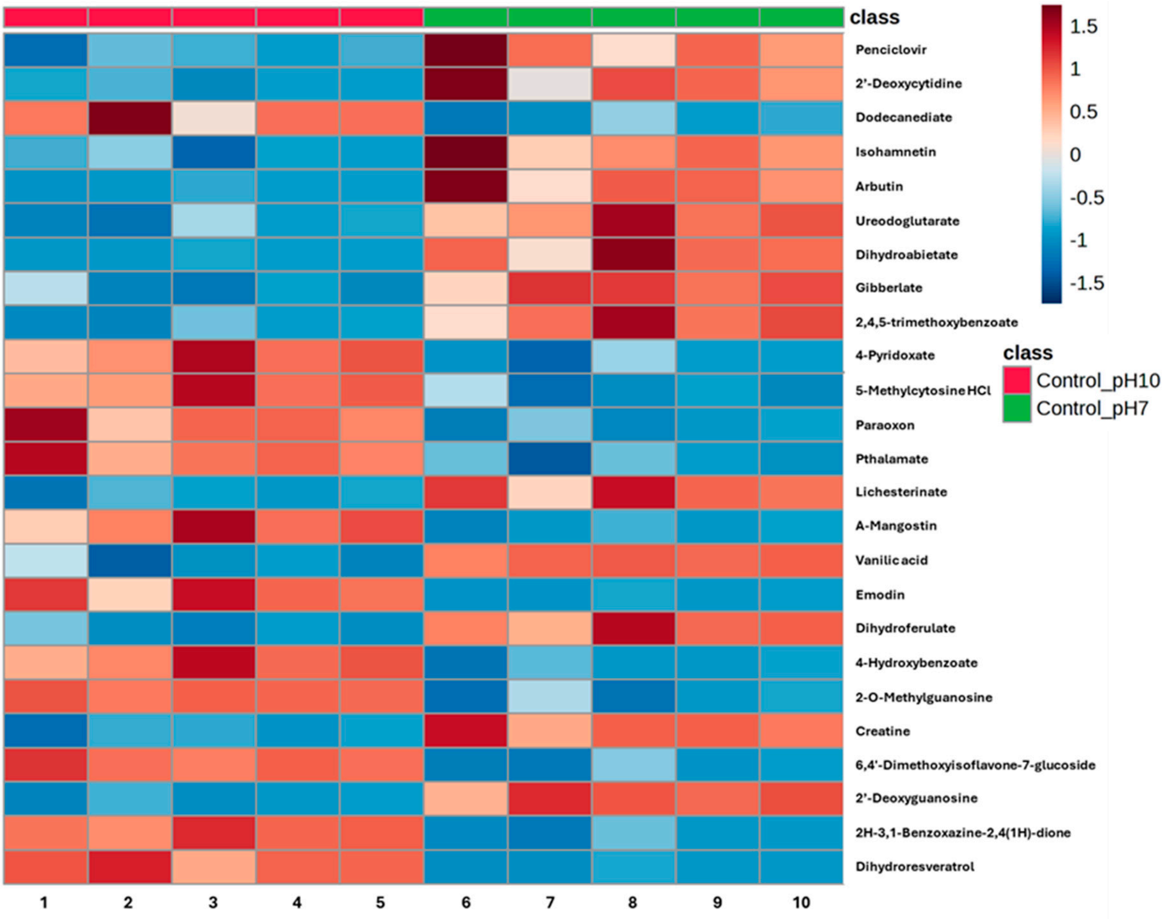

Supplementary Figure S1. Top 25 statistically significant (FDR adjusted p-value ≤ 0.05) metabolites that were found to be elevated or depleted at pH 7 and pH 10 conditions in the growth media, prior to microbial inoculation.

The appendix is an optional section that can contain details and data supplemental to the main text—for example, explanations of experimental details that would disrupt the flow of the main text but nonetheless remain crucial to understanding and reproducing the research shown; figures of replicates for experiments of which representative data is shown in the main text can be added here if brief, or as Supplementary data. Mathematical proofs of results not central to the paper can be added as an appendix.

Supplementary Table S1. Statistically significant metabolites in pre-fermented canola samples.

Metabolite	FC	p.ajusted
Dihydroresveratrol	4.4383	0.0001
6,4'-Dimethoxyisoflavone-7-glucoside	4.0361	0.0001
2'-Deoxyguanosine 3'-monophosphate	0.2492	0.0001

2H-3,1-Benzoxazine-2,4(1H)-dione	2.7271	0.0001
Creatine	0.4089	0.0004
4-hydroxybenzoate	5.5576	0.0005
2-o-methylguanosine	4.2408	0.0005
Dihydroferulic acid	0.1599	0.0006
Emodin	4.7845	0.0009
Alpha-mangostin	3.8568	0.0014
Lichesterinic acid	0.4458	0.0014
Phthalamic acid	2.2649	0.0015
Paraoxon	2.1969	0.0015
5-methylcytosine hydrocloride	2.8526	0.0024
Dehydroabietic acid	0.2018	0.0027
Gibberellic acid	0.3951	0.0027
2,4,5-trimethoxybenzoic acid	0.4154	0.0027
Ureidoglutaric acid	0.4710	0.0029
Arbutin	0.2020	0.0036
2'-deoxycytidine	0.1221	0.0073
Dodecanedioic acid	3.4694	0.0073
Isorhamnetin	0.3121	0.0073
1-methylguanosine	2.6246	0.0093
Genipin	0.0279	0.0109
D-Glucuronic acid	0.1604	0.0139
Ciprofibrate	2.3043	0.0139
N-Acetyl-DL-aspartic acid	2.1709	0.0144
Sodium phenylpyruvate	2.5160	0.0147
Avocadyne acetate	3.1231	0.0251
Silychristin	0.4136	0.0330
3,4-dihydroxybenzoate	2.1183	0.0330
Formyl-l-methionyl peptide	2.8713	0.0357
Dethiobiotin	2.1617	0.0362
Fallacinal	0.4615	0.0399
Santonin	0.4391	0.0402
Pseudocyphellarin A	5.4016	0.0475
3-Hydroxyglutaric acid	0.2221	0.0475
Citraconic acid	0.2431	0.0475
3-phenyllactic acid	3.5123	0.0475
2-Hydroxyisocaproic acid	3.0502	0.0475
Pseudo-anisatin	2.8293	0.0475
N-Methylglutamic acid	2.5791	0.0475
3-Hydroxycinnamic acid	11.5330	0.0499
DL-p-Hydroxyphenyllactic acid	8.3158	0.0499
N-Fructosyl pyroglutamate	2.7012	0.0499
3,4-Dihydroxy-L-phenylalanine	2.3771	0.0499
2-Hydroxyphenanzine-1-Carboxylic acid	2.2752	0.0499
3-hydroxybenzoate	2.1929	0.0499
Tricetin	0.4931	0.0499

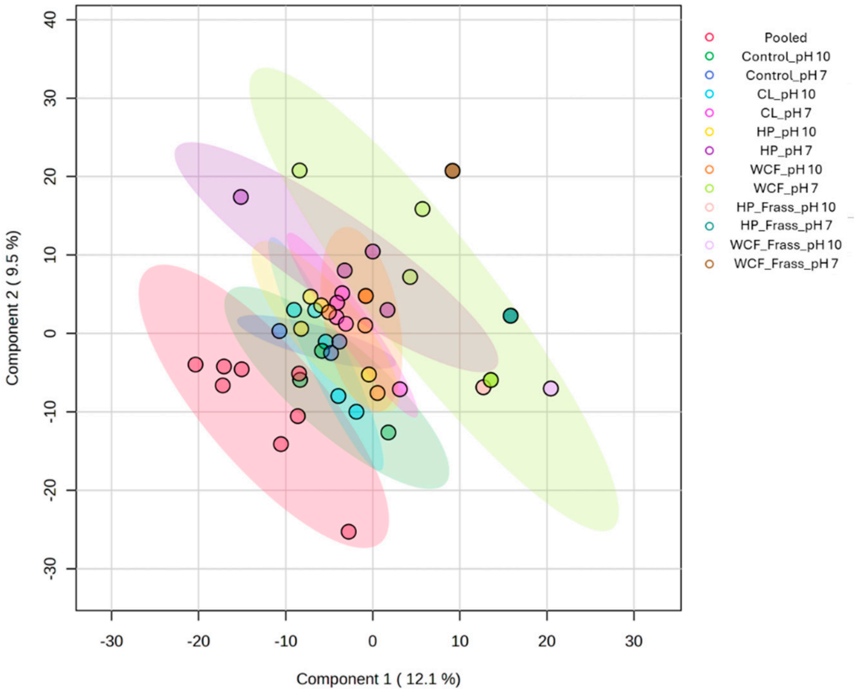

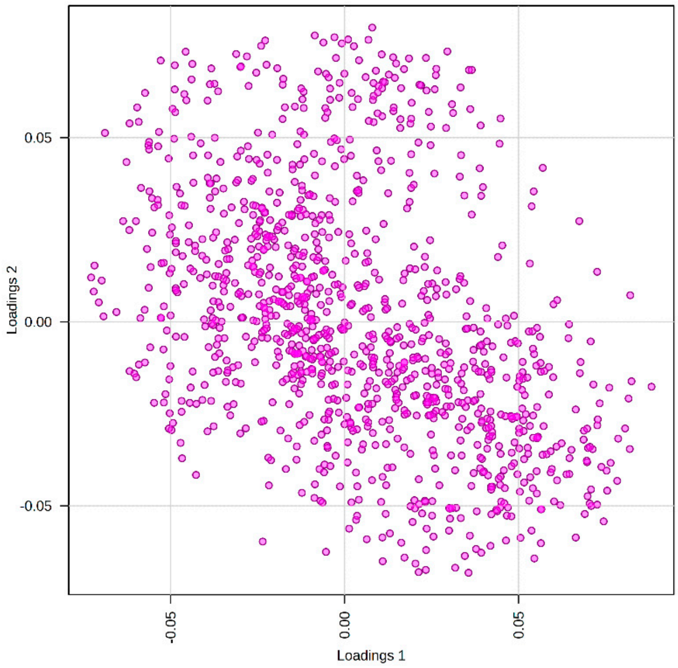

Supplementary Figure S2. Most impactful pathways (FDR adj. p-value ≤ 0.05) during the canola meal fermentation as analysed by the Pathway analysis toolbox of Metaboanalyst 6.0.

Supplementary Table S2. Statistically significant metabolites in pre-fermented canola samples.

Pathway (*Bombyx mori*)	Total Compounds	Hits	FDR	Impact
Purine metabolism	70	16	5.2889e-13	0.2054
Pyrimidine metabolism	37	10	1.368e-12	0.3851
Tyrosine metabolism	29	10	2.7761e-10	0.6676
Pentose phosphate pathway	24	10	1.0942e-08	0.4015
Alanine, aspartate and glutamate metabolism	21	14	3.3337e-08	0.9788
Glycine, serine and threonine metabolism	30	9	9.7783e-08	0.7329
Glutathione metabolism	26	8	2.2164e-07	0.4945
Inositol phosphate metabolism	29	3	4.2358e-07	0.0000
Ascorbate and aldarate metabolism	9	2	1.4718e-06	0.5238
Amino sugar and nucleotide sugar metabolism	35	9	1.5204e-06	0.2615
Nicotinate and nicotinamide metabolism	9	2	2.7272e-06	0.0377
Citrate cycle (TCA cycle)	20	8	2.7272e-06	0.4698
Lipoic acid metabolism	27	4	2.7272e-06	0.0019
Tryptophan metabolism	29	8	4.3429e-06	0.5275
Butanoate metabolism	14	6	4.3429e-06	0.1000
Fatty acid biosynthesis	43	3	6.808e-06	0.0168
Pyruvate metabolism	23	4	3.4818e-05	0.1849
Pentose and glucuronate interconversions	18	3	3.4818e-05	0.1923
Lysine degradation	28	5	3.4818e-05	0.2114
Ubiquinone and other terpenoid-quinone biosynthesis	19	5	4.4854e-05	0.0455
Thiamine metabolism	7	2	6.7907e-05	0.0000
beta-Alanine metabolism	16	4	9.5022e-05	0.0456
Glyoxylate and dicarboxylate metabolism	24	9	0.0002	0.3881
Arginine and proline metabolism	29	9	0.0003	0.3704
Arginine biosynthesis	13	9	0.0003	0.7954
Porphyrin metabolism	24	3	0.0003	0.0000
Phenylalanine metabolism	8	6	0.0004	0.7381
D-Amino acid metabolism	12	2	0.0009	0.0000
Glycerolipid metabolism	12	2	0.0010	0.1371
Propanoate metabolism	20	2	0.0013	0.0000
Glycolysis or Gluconeogenesis	26	5	0.0013	0.1792
Fructose and mannose metabolism	18	4	0.0014	0.3286
Pantothenate and CoA biosynthesis	17	4	0.0016	0.0000
Histidine metabolism	9	4	0.0028	0.4000
One carbon pool by folate	23	9	0.0028	0.4382
Vitamin B6 metabolism	8	3	0.0045	0.5000
Cysteine and methionine metabolism	34	11	0.0048	0.4826

Galactose metabolism	15	6	0.0086	0.3641
Terpenoid backbone biosynthesis	18	1	0.0088	0.0095
Glycerophospholipid metabolism	32	6	0.0117	0.2185
Riboflavin metabolism	4	1	0.0129	0.5000
Biotin metabolism	8	1	0.0143	0.0000
Starch and sucrose metabolism	14	4	0.0234	0.3343
alpha-Linolenic acid metabolism	8	1	0.0491	0.0000
Nitrogen metabolism	5	2	0.0491	0.0000
